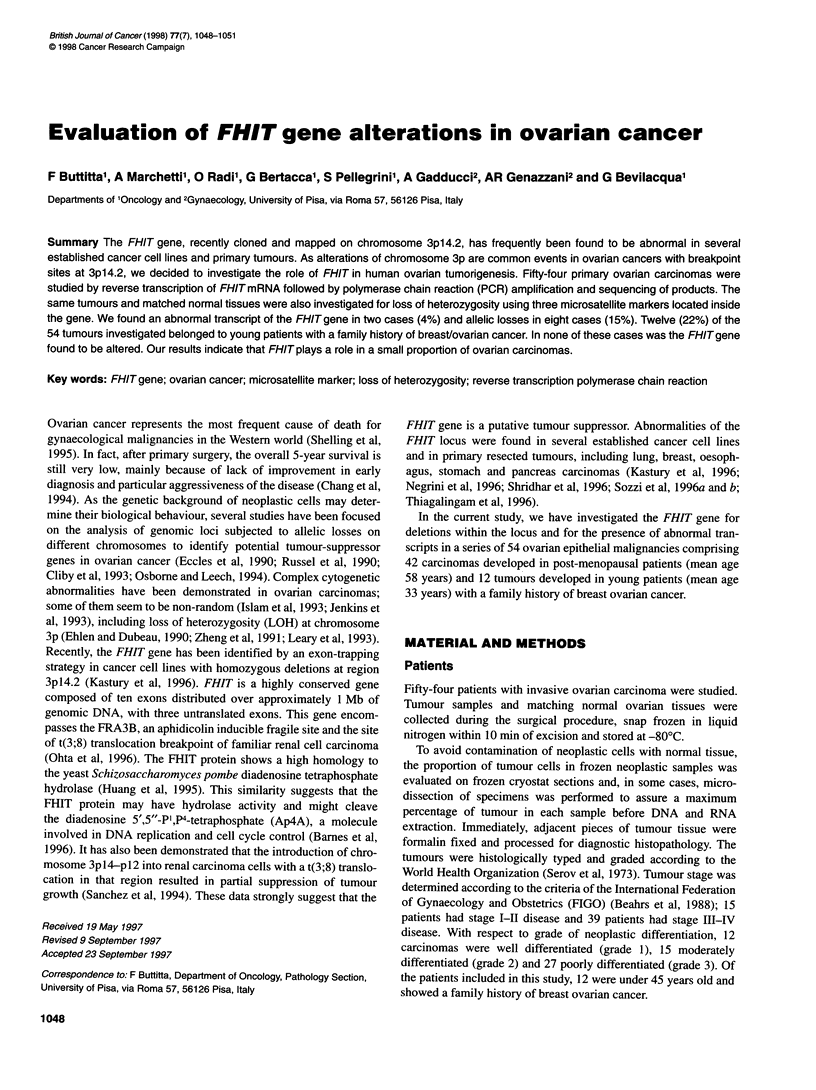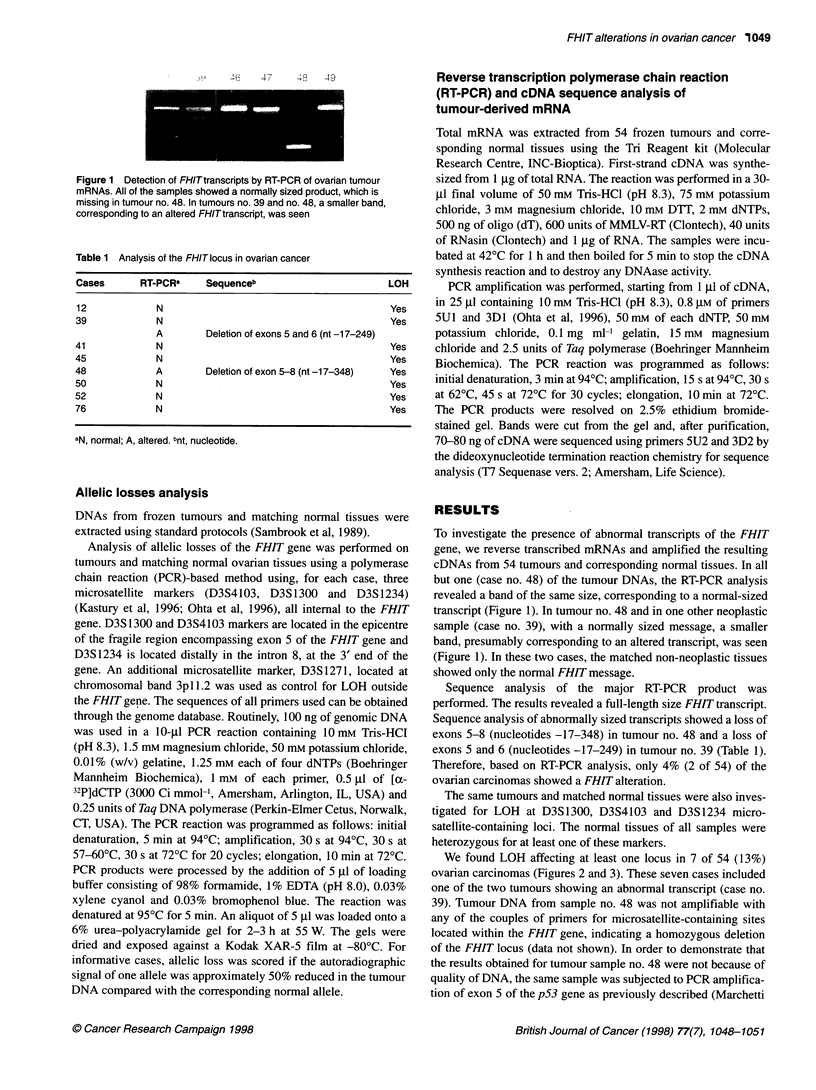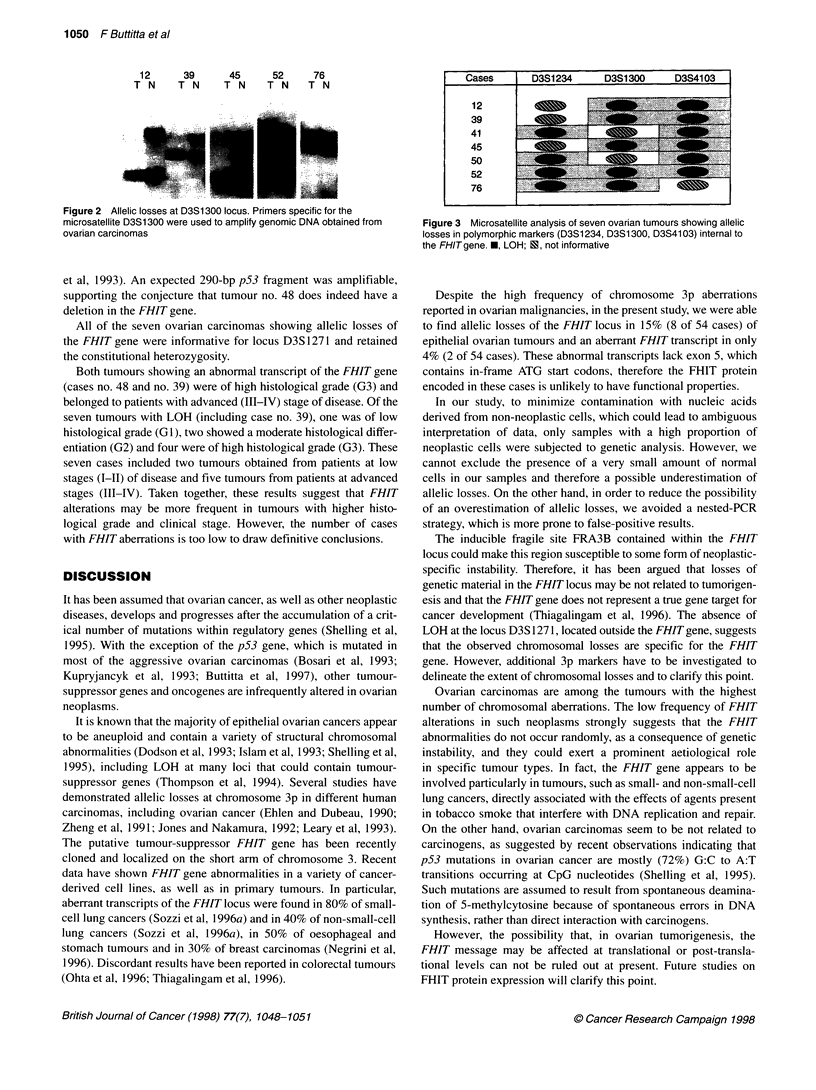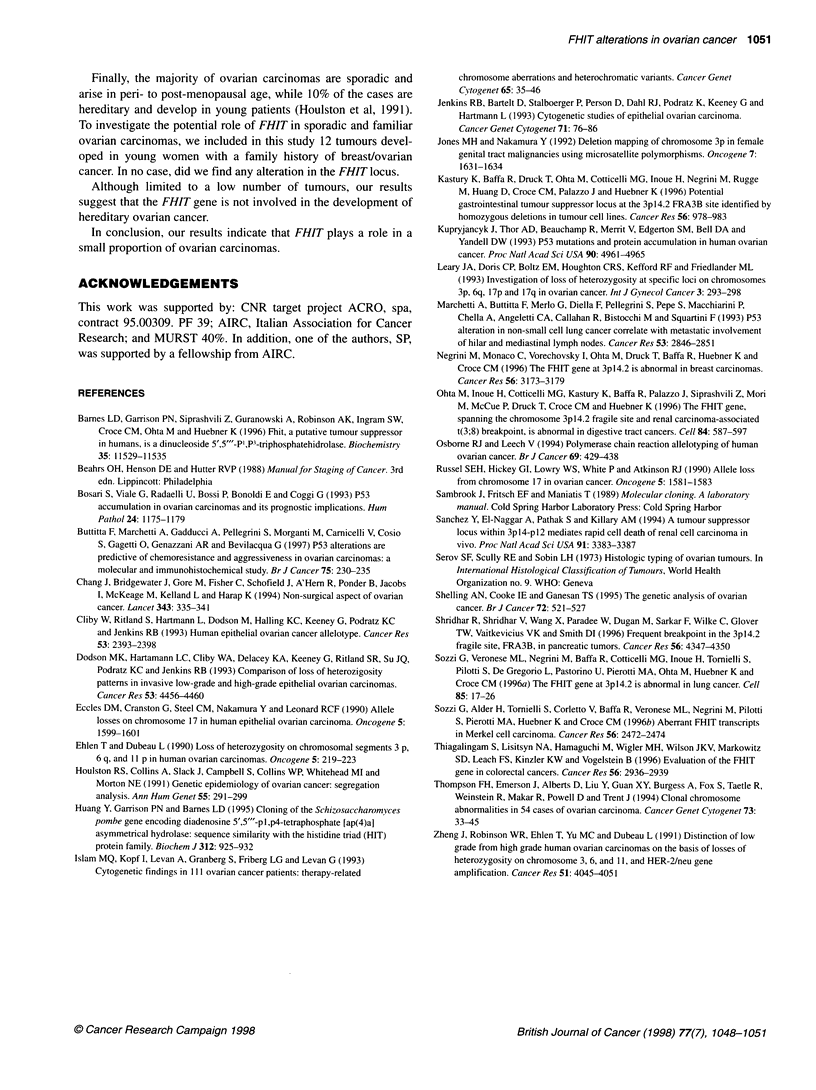# Evaluation of FHIT gene alterations in ovarian cancer.

**DOI:** 10.1038/bjc.1998.175

**Published:** 1998-04

**Authors:** F. Buttitta, A. Marchetti, O. Radi, G. Bertacca, S. Pellegrini, A. Gadducci, A. R. Genazzani, G. Bevilacqua

**Affiliations:** Department of Oncology, University of Pisa, Italy.

## Abstract

**Images:**


British Journal of Cancer (1998) 77(7), 1048–1051
© 1998 Cancer Research Campaign

### Evaluation of *FHIT* gene alterations in ovarian cancer

**F Buttitta[1], A Marchetti[1], O Radi[1], G Bertacca[1], S Pellegrini[1], A Gadducci[2], AR Genazzani[2] and G Bevilacqua[1]**

Departments of [1]Oncology and [2]Gynaecology, University of Pisa, via Roma 57, 56126 Pisa, Italy

**Summary** The *FHIT* gene, recently cloned and mapped on chromosome 3p14.2, has frequently been found to be abnormal in several established cancer cell lines and primary tumours. As alterations of chromosome 3p are common events in ovarian cancers with breakpoint sites at 3p14.2, we decided to investigate the role of *FHIT* in human ovarian tumorigenesis. Fifty-four primary ovarian carcinomas were studied by reverse transcription of *FHIT* mRNA followed by polymerase chain reaction (PCR) amplification and sequencing of products. The same tumours and matched normal tissues were also investigated for loss of heterozygosity using three microsatellite markers located inside the gene. We found an abnormal transcript of the *FHIT* gene in two cases (4%) and allelic losses in eight cases (15%). Twelve (22%) of the 54 tumours investigated belonged to young patients with a family history of breast/ovarian cancer. In none of these cases was the *FHIT* gene found to be altered. Our results indicate that *FHIT* plays a role in a small proportion of ovarian carcinomas.

**Key words:** *FHIT* gene; ovarian cancer; microsatellite marker; loss of heterozygosity; reverse transcription polymerase chain reaction

Ovarian cancer represents the most frequent cause of death for gynaecological malignancies in the Western world (Shelling et al, 1995). In fact, after primary surgery, the overall 5-year survival is still very low, mainly because of lack of improvement in early diagnosis and particular aggressiveness of the disease (Chang et al, 1994). As the genetic background of neoplastic cells may determine their biological behaviour, several studies have been focused on the analysis of genomic loci subjected to allelic losses on different chromosomes to identify potential tumour-suppressor genes in ovarian cancer (Eccles et al, 1990; Russel et al, 1990; Cliby et al, 1993; Osborne and Leech, 1994). Complex cytogenetic abnormalities have been demonstrated in ovarian carcinomas; some of them seem to be non-random (Islam et al, 1993; Jenkins et al, 1993), including loss of heterozygosity (LOH) at chromosome 3p (Ehlen and Dubeau, 1990; Zheng et al, 1991; Leary et al, 1993). Recently, the *FHIT* gene has been identified by an exon-trapping strategy in cancer cell lines with homozygous deletions at region 3p14.2 (Kastury et al, 1996). *FHIT* is a highly conserved gene composed of ten exons distributed over approximately 1 Mb of genomic DNA, with three untranslated exons. This gene encompasses the FRA3B, an aphidicolin inducible fragile site and the site of t(3;8) translocation breakpoint of familiar renal cell carcinoma (Ohta et al, 1996). The FHIT protein shows a high homology to the yeast *Schizosaccharomyces pombe* diadenosine tetraphosphate hydrolase (Huang et al, 1995). This similarity suggests that the FHIT protein may have hydrolase activity and might cleave the diadenosine 5′,5″-$P^1$,$P^4$-tetraphosphate (Ap4A), a molecule involved in DNA replication and cell cycle control (Barnes et al, 1996). It has also been demonstrated that the introduction of chromosome 3p14–p12 into renal carcinoma cells with a t(3;8) translocation in that region resulted in partial suppression of tumour growth (Sanchez et al, 1994). These data strongly suggest that the *FHIT* gene is a putative tumour suppressor. Abnormalities of the *FHIT* locus were found in several established cancer cell lines and in primary resected tumours, including lung, breast, oesophagus, stomach and pancreas carcinomas (Kastury et al, 1996; Negrini et al, 1996; Shridhar et al, 1996; Sozzi et al, 1996*a* and *b*; Thiagalingam et al, 1996).

In the current study, we have investigated the *FHIT* gene for deletions within the locus and for the presence of abnormal transcripts in a series of 54 ovarian epithelial malignancies comprising 42 carcinomas developed in post-menopausal patients (mean age 58 years) and 12 tumours developed in young patients (mean age 33 years) with a family history of breast ovarian cancer.

*Received 19 May 1997*
*Revised 9 September 1997*
*Accepted 23 September 1997*

*Correspondence to:* F Buttitta, Department of Oncology, Pathology Section, University of Pisa, via Roma 57, 56126 Pisa, Italy

### MATERIAL AND METHODS

### Patients

Fifty-four patients with invasive ovarian carcinoma were studied. Tumour samples and matching normal ovarian tissues were collected during the surgical procedure, snap frozen in liquid nitrogen within 10 min of excision and stored at –80°C.

To avoid contamination of neoplastic cells with normal tissue, the proportion of tumour cells in frozen neoplastic samples was evaluated on frozen cryostat sections and, in some cases, microdissection of specimens was performed to assure a maximum percentage of tumour in each sample before DNA and RNA extraction. Immediately, adjacent pieces of tumour tissue were formalin fixed and processed for diagnostic histopathology. The tumours were histologically typed and graded according to the World Health Organization (Serov et al, 1973). Tumour stage was determined according to the criteria of the International Federation of Gynaecology and Obstetrics (FIGO) (Beahrs et al, 1988); 15 patients had stage I–II disease and 39 patients had stage III–IV disease. With respect to grade of neoplastic differentiation, 12 carcinomas were well differentiated (grade 1), 15 moderately differentiated (grade 2) and 27 poorly differentiated (grade 3). Of the patients included in this study, 12 were under 45 years old and showed a family history of breast ovarian cancer.

**Figure 1** Detection of *FHIT* transcripts by RT-PCR of ovarian tumour mRNAs. All of the samples showed a normally sized product, which is missing in tumour no. 48. In tumours no. 39 and no. 48, a smaller band, corresponding to an altered *FHIT* transcript, was seen

**Table 1** Analysis of the *FHIT* locus in ovarian cancer

| Cases | RT-PCR[a] | Sequence[b] | LOH |
|---|---|---|---|
| 12 | N | | Yes |
| 39 | N | | Yes |
| | A | Deletion of exons 5 and 6 (nt –17–249) | |
| 41 | N | | Yes |
| 45 | N | | Yes |
| 48 | A | Deletion of exon 5–8 (nt –17–348) | Yes |
| 50 | N | | Yes |
| 52 | N | | Yes |
| 76 | N | | Yes |

[a]N, normal; A, altered. [b]nt, nucleotide.

### Allelic losses analysis

DNAs from frozen tumours and matching normal tissues were extracted using standard protocols (Sambrook et al, 1989).

Analysis of allelic losses of the *FHIT* gene was performed on tumours and matching normal ovarian tissues using a polymerase chain reaction (PCR)-based method using, for each case, three microsatellite markers (D3S4103, D3S1300 and D3S1234) (Kastury et al, 1996; Ohta et al, 1996), all internal to the *FHIT* gene. D3S1300 and D3S4103 markers are located in the epicentre of the fragile region encompassing exon 5 of the *FHIT* gene and D3S1234 is located distally in the intron 8, at the 3′ end of the gene. An additional microsatellite marker, D3S1271, located at chromosomal band 3p11.2 was used as control for LOH outside the *FHIT* gene. The sequences of all primers used can be obtained through the genome database. Routinely, 100 ng of genomic DNA was used in a 10-μl PCR reaction containing 10 mM Tris-HCl (pH 8.3), 1.5 mM magnesium chloride, 50 mM potassium chloride, 0.01% (w/v) gelatine, 1.25 mM each of four dNTPs (Boehringer Mannheim Biochemica), 1 mM of each primer, 0.5 μl of [α-$^{32}$P]dCTP (3000 Ci mmol$^{-1}$, Amersham, Arlington, IL, USA) and 0.25 units of *Taq* DNA polymerase (Perkin-Elmer Cetus, Norwalk, CT, USA). The PCR reaction was programmed as follows: initial denaturation, 5 min at 94°C; amplification, 30 s at 94°C, 30 s at 57–60°C, 30 s at 72°C for 20 cycles; elongation, 10 min at 72°C. PCR products were processed by the addition of 5 μl of loading buffer consisting of 98% formamide, 1% EDTA (pH 8.0), 0.03% xylene cyanol and 0.03% bromophenol blue. The reaction was denatured at 95°C for 5 min. An aliquot of 5 μl was loaded onto a 6% urea–polyacrylamide gel for 2–3 h at 55 W. The gels were dried and exposed against a Kodak XAR-5 film at -80°C. For informative cases, allelic loss was scored if the autoradiographic signal of one allele was approximately 50% reduced in the tumour DNA compared with the corresponding normal allele.

### Reverse transcription polymerase chain reaction (RT-PCR) and cDNA sequence analysis of tumour-derived mRNA

Total mRNA was extracted from 54 frozen tumours and corresponding normal tissues using the Tri Reagent kit (Molecular Research Centre, INC-Bioptica). First-strand cDNA was synthesized from 1 μg of total RNA. The reaction was performed in a 30-μl final volume of 50 mM Tris-HCl (pH 8.3), 75 mM potassium chloride, 3 mM magnesium chloride, 10 mM DTT, 2 mM dNTPs, 500 ng of oligo (dT), 600 units of MMLV-RT (Clontech), 40 units of RNasin (Clontech) and 1 μg of RNA. The samples were incubated at 42°C for 1 h and then boiled for 5 min to stop the cDNA synthesis reaction and to destroy any DNAase activity.

PCR amplification was performed, starting from 1 μl of cDNA, in 25 μl containing 10 mM Tris-HCl (pH 8.3), 0.8 μM of primers 5U1 and 3D1 (Ohta et al, 1996), 50 mM of each dNTP, 50 mM potassium chloride, 0.1 mg ml$^{-1}$ gelatin, 15 mM magnesium chloride and 2.5 units of *Taq* polymerase (Boehringer Mannheim Biochemica). The PCR reaction was programmed as follows: initial denaturation, 3 min at 94°C; amplification, 15 s at 94°C, 30 s at 62°C, 45 s at 72°C for 30 cycles; elongation, 10 min at 72°C. The PCR products were resolved on 2.5% ethidium bromide-stained gel. Bands were cut from the gel and, after purification, 70–80 ng of cDNA were sequenced using primers 5U2 and 3D2 by the dideoxynucleotide termination reaction chemistry for sequence analysis (T7 Sequenase vers. 2; Amersham, Life Science).

### RESULTS

To investigate the presence of abnormal transcripts of the *FHIT* gene, we reverse transcribed mRNAs and amplified the resulting cDNAs from 54 tumours and corresponding normal tissues. In all but one (case no. 48) of the tumour DNAs, the RT-PCR analysis revealed a band of the same size, corresponding to a normal-sized transcript (Figure 1). In tumour no. 48 and in one other neoplastic sample (case no. 39), with a normally sized message, a smaller band, presumably corresponding to an altered transcript, was seen (Figure 1). In these two cases, the matched non-neoplastic tissues showed only the normal *FHIT* message.

Sequence analysis of the major RT-PCR product was performed. The results revealed a full-length size *FHIT* transcript. Sequence analysis of abnormally sized transcripts showed a loss of exons 5–8 (nucleotides –17–348) in tumour no. 48 and a loss of exons 5 and 6 (nucleotides –17–249) in tumour no. 39 (Table 1). Therefore, based on RT-PCR analysis, only 4% (2 of 54) of the ovarian carcinomas showed a *FHIT* alteration.

The same tumours and matched normal tissues were also investigated for LOH at D3S1300, D3S4103 and D3S1234 microsatellite-containing loci. The normal tissues of all samples were heterozygous for at least one of these markers.

We found LOH affecting at least one locus in 7 of 54 (13%) ovarian carcinomas (Figures 2 and 3). These seven cases included one of the two tumours showing an abnormal transcript (case no. 39). Tumour DNA from sample no. 48 was not amplifiable with any of the couples of primers for microsatellite-containing sites located within the *FHIT* gene, indicating a homozygous deletion of the *FHIT* locus (data not shown). In order to demonstrate that the results obtained for tumour sample no. 48 were not because of quality of DNA, the same sample was subjected to PCR amplification of exon 5 of the *p53* gene as previously described (Marchetti

© Cancer Research Campaign 1998

**Figure 2** Allelic losses at D3S1300 locus. Primers specific for the microsatellite D3S1300 were used to amplify genomic DNA obtained from ovarian carcinomas

**Figure 3** Microsatellite analysis of seven ovarian tumours showing allelic losses in polymorphic markers (D3S1234, D3S1300, D3S4103) internal to the *FHIT* gene. ■, LOH; ▧, not informative et al, 1993). An expected 290-bp *p53* fragment was amplifiable, supporting the conjecture that tumour no. 48 does indeed have a deletion in the *FHIT* gene.

All of the seven ovarian carcinomas showing allelic losses of the *FHIT* gene were informative for locus D3S1271 and retained the constitutional heterozygosity.

Both tumours showing an abnormal transcript of the *FHIT* gene (cases no. 48 and no. 39) were of high histological grade (G3) and belonged to patients with advanced (III–IV) stage of disease. Of the seven tumours with LOH (including case no. 39), one was of low histological grade (G1), two showed a moderate histological differentiation (G2) and four were of high histological grade (G3). These seven cases included two tumours obtained from patients at low stages (I–II) of disease and five tumours from patients at advanced stages (III–IV). Taken together, these results suggest that *FHIT* alterations may be more frequent in tumours with higher histological grade and clinical stage. However, the number of cases with *FHIT* aberrations is too low to draw definitive conclusions.

### DISCUSSION

It has been assumed that ovarian cancer, as well as other neoplastic diseases, develops and progresses after the accumulation of a critical number of mutations within regulatory genes (Shelling et al, 1995). With the exception of the *p53* gene, which is mutated in most of the aggressive ovarian carcinomas (Bosari et al, 1993; Kupryjancyk et al, 1993; Buttitta et al, 1997), other tumour-suppressor genes and oncogenes are infrequently altered in ovarian neoplasms.

It is known that the majority of epithelial ovarian cancers appear to be aneuploid and contain a variety of structural chromosomal abnormalities (Dodson et al, 1993; Islam et al, 1993; Shelling et al, 1995), including LOH at many loci that could contain tumour-suppressor genes (Thompson et al, 1994). Several studies have demonstrated allelic losses at chromosome 3p in different human carcinomas, including ovarian cancer (Ehlen and Dubeau, 1990; Zheng et al, 1991; Jones and Nakamura, 1992; Leary et al, 1993). The putative tumour-suppressor *FHIT* gene has been recently cloned and localized on the short arm of chromosome 3. Recent data have shown *FHIT* gene abnormalities in a variety of cancer-derived cell lines, as well as in primary tumours. In particular, aberrant transcripts of the *FHIT* locus were found in 80% of small-cell lung cancers (Sozzi et al, 1996*a*) and in 40% of non-small-cell lung cancers (Sozzi et al, 1996*a*), in 50% of oesophageal and stomach tumours and in 30% of breast carcinomas (Negrini et al, 1996). Discordant results have been reported in colorectal tumours (Ohta et al, 1996; Thiagalingam et al, 1996).

Despite the high frequency of chromosome 3p aberrations reported in ovarian malignancies, in the present study, we were able to find allelic losses of the *FHIT* locus in 15% (8 of 54 cases) of epithelial ovarian tumours and an aberrant *FHIT* transcript in only 4% (2 of 54 cases). These abnormal transcripts lack exon 5, which contains in-frame ATG start codons, therefore the FHIT protein encoded in these cases is unlikely to have functional properties.

In our study, to minimize contamination with nucleic acids derived from non-neoplastic cells, which could lead to ambiguous interpretation of data, only samples with a high proportion of neoplastic cells were subjected to genetic analysis. However, we cannot exclude the presence of a very small amount of normal cells in our samples and therefore a possible underestimation of allelic losses. On the other hand, in order to reduce the possibility of an overestimation of allelic losses, we avoided a nested-PCR strategy, which is more prone to false-positive results.

The inducible fragile site FRA3B contained within the *FHIT* locus could make this region susceptible to some form of neoplastic-specific instability. Therefore, it has been argued that losses of genetic material in the *FHIT* locus may be not related to tumorigenesis and that the *FHIT* gene does not represent a true gene target for cancer development (Thiagalingam et al, 1996). The absence of LOH at the locus D3S1271, located outside the *FHIT* gene, suggests that the observed chromosomal losses are specific for the *FHIT* gene. However, additional 3p markers have to be investigated to delineate the extent of chromosomal losses and to clarify this point.

Ovarian carcinomas are among the tumours with the highest number of chromosomal aberrations. The low frequency of *FHIT* alterations in such neoplasms strongly suggests that the *FHIT* abnormalities do not occur randomly, as a consequence of genetic instability, and they could exert a prominent aetiological role in specific tumour types. In fact, the *FHIT* gene appears to be involved particularly in tumours, such as small- and non-small-cell lung cancers, directly associated with the effects of agents present in tobacco smoke that interfere with DNA replication and repair. On the other hand, ovarian carcinomas seem to be not related to carcinogens, as suggested by recent observations indicating that *p53* mutations in ovarian cancer are mostly (72%) G:C to A:T transitions occurring at CpG nucleotides (Shelling et al, 1995). Such mutations are assumed to result from spontaneous deamination of 5-methylcytosine because of spontaneous errors in DNA synthesis, rather than direct interaction with carcinogens.

However, the possibility that, in ovarian tumorigenesis, the *FHIT* message may be affected at translational or post-translational levels can not be ruled out at present. Future studies on FHIT protein expression will clarify this point.

*© Cancer Research Campaign 1998*

Finally, the majority of ovarian carcinomas are sporadic and arise in peri- to post-menopausal age, while 10% of the cases are hereditary and develop in young patients (Houlston et al, 1991). To investigate the potential role of *FHIT* in sporadic and familiar ovarian carcinomas, we included in this study 12 tumours developed in young women with a family history of breast/ovarian cancer. In no case, did we find any alteration in the *FHIT* locus.

Although limited to a low number of tumours, our results suggest that the *FHIT* gene is not involved in the development of hereditary ovarian cancer.

In conclusion, our results indicate that *FHIT* plays a role in a small proportion of ovarian carcinomas.

### ACKNOWLEDGEMENTS

This work was supported by: CNR target project ACRO, spa, contract 95.00309. PF 39; AIRC, Italian Association for Cancer Research; and MURST 40%. In addition, one of the authors, SP, was supported by a fellowship from AIRC.

### REFERENCES

Barnes LD, Garrison PN, Siprashvili Z, Guranowski A, Robinson AK, Ingram SW, Croce CM, Ohta M and Huebner K (1996) Fhit, a putative tumour suppressor in humans, is a dinucleoside 5′,5‴-$P^1$,$P^3$-triphosphatehidrolase. *Biochemistry* **35**: 11529–11535

Beahrs OH, Henson DE and Hutter RVP (1988) *Manual for Staging of Cancer*. 3rd edn. Lippincott: Philadelphia

Bosari S, Viale G, Radaelli U, Bossi P, Bonoldi E and Coggi G (1993) P53 accumulation in ovarian carcinomas and its prognostic implications. *Hum Pathol* **24**: 1175–1179

Buttitta F, Marchetti A, Gadducci A, Pellegrini S, Morganti M, Carnicelli V, Cosio S, Gagetti O, Genazzani AR and Bevilacqua G (1997) P53 alterations are predictive of chemoresistance and aggressiveness in ovarian carcinomas: a molecular and immunohistochemical study. *Br J Cancer* **75**: 230–235

Chang J, Bridgewater J, Gore M, Fisher C, Schofield J, A'Hern R, Ponder B, Jacobs I, McKeage M, Kelland L and Harap K (1994) Non-surgical aspect of ovarian cancer. *Lancet* **343**: 335–341

Cliby W, Ritland S, Hartmann L, Dodson M, Halling KC, Keeney G, Podratz KC and Jenkins RB (1993) Human epithelial ovarian cancer allelotype. *Cancer Res* **53**: 2393–2398

Dodson MK, Hartamann LC, Cliby WA, Delacey KA, Keeney G, Ritland SR, Su JQ, Podratz KC and Jenkins RB (1993) Comparison of loss of heterozigosity patterns in invasive low-grade and high-grade epithelial ovarian carcinomas. *Cancer Res* **53**: 4456–4460

Eccles DM, Cranston G, Steel CM, Nakamura Y and Leonard RCF (1990) Allele losses on chromosome 17 in human epithelial ovarian carcinoma. *Oncogene* **5**: 1599–1601

Ehlen T and Dubeau L (1990) Loss of heterozygosity on chromosomal segments 3 p, 6 q, and 11 p in human ovarian carcinomas. *Oncogene* **5**: 219–223

Houlston RS, Collins A, Slack J, Campbell S, Collins WP, Whitehead MI and Morton NE (1991) Genetic epidemiology of ovarian cancer: segregation analysis. *Ann Hum Genet* **55**: 291–299

Huang Y, Garrison PN and Barnes LD (1995) Cloning of the *Schizosaccharomyces pombe* gene encoding diadenosine 5′,5‴-p1,p4-tetraphosphate [ap(4)a] asymmetrical hydrolase: sequence similarity with the histidine triad (HIT) protein family. *Biochem J* **312**: 925–932

Islam MQ, Kopf I, Levan A, Granberg S, Friberg LG and Levan G (1993) Cytogenetic findings in 111 ovarian cancer patients: therapy-related chromosome aberrations and heterochromatic variants. *Cancer Genet Cytogenet* **65**: 35–46

Jenkins RB, Bartelt D, Stalboerger P, Person D, Dahl RJ, Podratz K, Keeney G and Hartmann L (1993) Cytogenetic studies of epithelial ovarian carcinoma. *Cancer Genet Cytogenet* **71**: 76–86

Jones MH and Nakamura Y (1992) Deletion mapping of chromosome 3p in female genital tract malignancies using microsatellite polymorphisms. *Oncogene* **7**: 1631–1634

Kastury K, Baffa R, Druck T, Ohta M, Cotticelli MG, Inoue H, Negrini M, Rugge M, Huang D, Croce CM, Palazzo J and Huebner K (1996) Potential gastrointestinal tumour suppressor locus at the 3p14.2 FRA3B site identified by homozygous deletions in tumour cell lines. *Cancer Res* **56**: 978–983

Kupryjancyk J, Thor AD, Beauchamp R, Merrit V, Edgerton SM, Bell DA and Yandell DW (1993) P53 mutations and protein accumulation in human ovarian cancer. *Proc Natl Acad Sci USA* **90**: 4961–4965

Leary JA, Doris CP, Boltz EM, Houghton CRS, Kefford RF and Friedlander ML (1993) Investigation of loss of heterozygosity at specific loci on chromosomes 3p, 6q, 17p and 17q in ovarian cancer. *Int J Gynecol Cancer* **3**: 293–298

Marchetti A, Buttitta F, Merlo G, Diella F, Pellegrini S, Pepe S, Macchiarini P, Chella A, Angeletti CA, Callahan R, Bistocchi M and Squartini F (1993) P53 alteration in non-small cell lung cancer correlate with metastatic involvement of hilar and mediastinal lymph nodes. *Cancer Res* **53**: 2846–2851

Negrini M, Monaco C, Vorechovsky I, Ohta M, Druck T, Baffa R, Huebner K and Croce CM (1996) The FHIT gene at 3p14.2 is abnormal in breast carcinomas. *Cancer Res* **56**: 3173–3179

Ohta M, Inoue H, Cotticelli MG, Kastury K, Baffa R, Palazzo J, Siprashvili Z, Mori M, McCue P, Druck T, Croce CM and Huebner K (1996) The FHIT gene, spanning the chromosome 3p14.2 fragile site and renal carcinoma-associated t(3;8) breakpoint, is abnormal in digestive tract cancers. *Cell* **84**: 587–597

Osborne RJ and Leech V (1994) Polymerase chain reaction allelotyping of human ovarian cancer. *Br J Cancer* **69**: 429–438

Russel SEH, Hickey GI, Lowry WS, White P and Atkinson RJ (1990) Allele loss from chromosome 17 in ovarian cancer. *Oncogene* **5**: 1581–1583

Sambrook J, Fritsch EF and Maniatis T (1989) *Molecular cloning. A laboratory manual*. Cold Spring Harbor Laboratory Press: Cold Spring Harbor

Sanchez Y, El-Naggar A, Pathak S and Killary AM (1994) A tumour suppressor locus within 3p14-p12 mediates rapid cell death of renal cell carcinoma in vivo. *Proc Natl Acad Sci USA* **91**: 3383–3387

Serov SF, Scully RE and Sobin LH (1973) Histologic typing of ovarian tumours. In *International Histological Classification of Tumours*, World Health Organization no. 9. WHO: Geneva

Shelling AN, Cooke IE and Ganesan TS (1995) The genetic analysis of ovarian cancer. *Br J Cancer* **72**: 521–527

Shridhar R, Shridhar V, Wang X, Paradee W, Dugan M, Sarkar F, Wilke C, Glover TW, Vaitkevicius VK and Smith DI (1996) Frequent breakpoint in the 3p14.2 fragile site, FRA3B, in pancreatic tumors. *Cancer Res* **56**: 4347–4350

Sozzi G, Veronese ML, Negrini M, Baffa R, Cotticelli MG, Inoue H, Tornielli S, Pilotti S, De Gregorio L, Pastorino U, Pierotti MA, Ohta M, Huebner K and Croce CM (1996*a*) The FHIT gene at 3p14.2 is abnormal in lung cancer. *Cell* **85**: 17–26

Sozzi G, Alder H, Tornielli S, Corletto V, Baffa R, Veronese ML, Negrini M, Pilotti S, Pierotti MA, Huebner K and Croce CM (1996*b*) Aberrant FHIT transcripts in Merkel cell carcinoma. *Cancer Res* **56**: 2472–2474

Thiagalingam S, Lisitsyn NA, Hamaguchi M, Wigler MH, Wilson JKV, Markowitz SD, Leach FS, Kinzler KW and Vogelstein B (1996) Evaluation of the FHIT gene in colorectal cancers. *Cancer Res* **56**: 2936–2939

Thompson FH, Emerson J, Alberts D, Liu Y, Guan XY, Burgess A, Fox S, Taetle R, Weinstein R, Makar R, Powell D and Trent J (1994) Clonal chromosome abnormalities in 54 cases of ovarian carcinoma. *Cancer Genet Cytogenet* **73**: 33–45

Zheng J, Robinson WR, Ehlen T, Yu MC and Dubeau L (1991) Distinction of low grade from high grade human ovarian carcinomas on the basis of losses of heterozygosity on chromosome 3, 6, and 11, and HER-2/neu gene amplification. *Cancer Res* **51**: 4045–4051

© Cancer Research Campaign 1998